# Epidemiological characteristics of heat-related illness: a nationwide study in Taiwan

**DOI:** 10.1186/s12889-025-24344-1

**Published:** 2025-09-24

**Authors:** Wan-Yin Kuo, Chien-Cheng Huang, Chi-An Chen, Chung-Han Ho, Chien-Chin Hsu, Hung-Jung Lin, Shih-Bin Su, Jhi-Joung Wang, How-Ran Guo

**Affiliations:** 1 Department of Emergency Medicine, Chi Mei Medical Center, 901 Zhonghua Road, Yongkang Dist., Tainan, 71004 Taiwan; 2 Department of Occupational Medicine, Chi Mei Medical Center, 901 Zhonghua Road, Yongkang Dist., Tainan, 71004 Taiwan; 3 Department of Environmental and Occupational Health, College of Medicine, National Cheng Kung University, 138 Shengli Road, Tainan, 70428 Taiwan; 4 School of Medicine, College of Medicine, National Sun Yat-sen University, 70 Lienhai Road, Kaohsiung, 804201 Taiwan; 5 Department of Emergency Medicine, Kaohsiung Medical University, 100 Shiquan 1st Road, Sanmin Dist., Kaohsiung, 807378 Taiwan; 6 Department of Medical Research, Chi Mei Medical Center, 901 Zhonghua Road, Yongkang Dist., Tainan, 71004 Taiwan; 7 Department of Information Management, Southern Taiwan University of Science and Technology, 1 Nantai Street, Yongkang Dist., Tainan, 71005 Taiwan; 8 Department of Emergency Medicine, Taipei Medical University, 250 Wuxing Street, Xinyi Dist., Taipei, 11031 Taiwan; 9 Department of Leisure, Recreation and Tourism Management, Southern Taiwan University of Science and Technology, 1 Nantai Street, Yongkang Dist., Tainan, 71005 Taiwan; 10 Department of Medical Research, Chi Mei Medical Center, Liouying, 201 Taikang, Liuying Dist., Tainan, 73657 Taiwan; 11 Department of Anesthesiology, National Defense Medical Center, 161 Minquan E. Road Section 6, Neihu Dist., Taipei, 11490 Taiwan; 12 Department of Occupational and Environmental Medicine, National Cheng Kung University Hospital, 138 Shengli Road, Tainan, 70428 Taiwan

**Keywords:** Epidemiology, Heat exhaustion, Heat related illness, Heat stroke, Heat syncope, Taiwan

## Abstract

**Background:**

Heat-related illness (HRI) is expected to occur more frequently and become a prominent issue worldwide in the context of global warming and climate change. Previous epidemiological studies of HRI were generally limited to selected populations or specific settings. The objective of this study was to characterize the epidemiological characteristics of HRI in a general population at the national level to fill the data gaps.

**Methods:**

Using the National Health Insurance Research Database, we identified all HRI patients in Taiwan between 2000 and 2018. We described the epidemiological characteristics of the patients and evaluated the differences between the two sexes. In addition, we evaluated the mortality rates of different types of HRI.

**Results:**

We identified 101,614 HRI patients, and male patients constituted the majority (56.2%). The mean age was 48.2 years, and most of the patients were between 20 and 44 years old (44.8%). In comparison with female patients, male patients were younger (46.4 vs. 50.5 years, *p* < 0.001) and more likely to receive treatment in hospitals (51.6% vs. 25.3%, *p* < 0.001). Among HRI, heat stroke was the most common diagnosis and had the highest mortality rate. The 7-day, 1-month, and 3-month mortality rates in heat stroke patients were 0.5%, 0.7% and 1.0%, respectively.

**Conclusions:**

In Taiwan, patients with HRI are more likely to males and between 20 and 44 years old. Male patients were younger and more likely to receive treatment in hospitals. Heat stroke was the most common HRI and had the highest mortality rate, which calls for establishment of the prevention and treatment strategies.

## Background

Rising global temperatures caused by climate change have profound effects on human health. Global temperatures have risen by approximately 1 °C compared to pre-industrial levels, with some regions experiencing increases of more than 2 °C [1]. It was estimated that vulnerable populations were exposed to an additional 475 million heatwave events in 2019 alone, leading to increased morbidity and mortality [2]. As temperatures continue to rise and extreme heat events grow in frequency and intensity, heat-related illness (HRI) is expected to occur more frequently and pose a major public health concern [3, 4].

HRI, disorders resulted from hyperthermia, is marked by a pathological elevation in core body temperature exceeding the thermoregulatory capacity [5]. Hyperthermia, which arises from environmental or exertional heat exposure, occurs when heat production exceeds the body’s ability to dissipate heat through mechanisms such as radiation, convection, and evaporation [6]. Prolonged hyperthermia can transition from a compensable phase to a noncompensable phase, resulting in systemic inflammatory responses, tissue injury, and multiorgan dysfunction [7, 8]. Recent studies have highlighted key contributors to the progression of hyperthermia, including intestinal ischemia, endotoxemia, and the release of inflammatory cytokines, which exacerbate systemic damages [6, 7]. These pathophysiologic changes underscore the severity of hyperthermia and its potential to progress to life-threatening conditions such as heat stroke.

​HRI comprises a broad spectrum of diseases ranging from mild illnesses such as heat cramp and heat edema, to more severe conditions including heat syncope and heat exhaustion, to the fatal condition of heat stroke [8, 9]. Heat stroke may lead to multiorgan failure and is a life-threatening condition with a high mortality rate if left untreated [4]. HRI may be caused by physical exercise, passive environmental exposure, or both [10]. Risk factors underlying HRI include age extremes, male sex, environmental factors, occupational exposure, and medical conditions such as cardiovascular diseases, diabetes mellitus, mental and pulmonary illnesses [4, 7, 9, 10]. Supportive care, rapid cooling measures, and rehydration with oral or intravenous fluids are the mainstay of treatments for HRI [9–11]. Mild to moderate HRI can be treated and discharged from the emergency departments (EDs). However, heat stroke patients generally require hospitalization or admission to intensive care unit [9, 10].

In the United States, heat-related injuries constitute the most common cause of environmental exposure-related injuries [12]. The Center for Disease Control and Prevention (CDC) reported a total of 3,066 heat-related deaths occurred in the United States during 2018–2020 [13]. The incidence of HRI was found to be 1.2 cases per 100,000 athletic exposures in high school athletes [14] and 1.77 cases per 1,000 person-years in Armed Forces [15]. An approximate average rate of ED visits for summertime HRI was 5 per 10,000 during 2006–2010 [16]. A recent study on an annual and national scale demonstrated the annual incidence rate of ED visits for HRI was 32.34 per 100,000 population in 2018, with an increase by an average of 2.85% per year from 2009 [17]. In Japan, deaths due to HRI occurred at a rate of about 500 per year since 2010 [18]. Previous epidemiological studies of HRI were generally limited to selected populations or specific settings (e.g., prehospital care, emergency department, and hospitalization). Therefore, the results might be biased or have limited use. Thus, we conducted a study to assess the incidence, demographic characteristics, and outcomes for HRI in the general population at the national level to fill the data gaps.

## Methods

### Data source

We used the National Health Insurance Research Database (NHIRD) to conduct this study, which contains registration files and original claim data for reimbursement from the National Health Insurance (NHI) program of Taiwan. The NHI was established in 1995 and had enrolled more than 99.9% of Taiwanese citizens in 2014 [19]. The NHIRD is one of the largest administrative health care databases in the world, which includes inpatient and outpatient data, prescribed medications, intervention procedures and the diagnoses of each claim that were coded by International Classification of Diseases, Ninth Revision, Clinical Modification (ICD-9-CM) or the International Classification of Diseases, Tenth Revision (ICD-10). NHIRD have been used for health care research, generating evidence to support clinical decisions and healthcare policy-making [20].

### Identification of HRI patients and definitions of study variables

In this cross-sectional study, we identified patients aged 20 and above with a new diagnosis of HRI, determined by the ICD-9-CM code of 992 or ICD-10 code of T67, between January 1, 2000 and December 31, 2018 for hospitalization, emergency department care, or outpatient department care. Among the HRI patients, we further identified those with heat stroke (ICD-9-CM: 992.0 or ICD-10: T67.0), heat exhaustion (ICD-9-CM: 992.3–992.5 or ICD-10: T67.3–T67.5), heat syncope (ICD-9-CM: 992.1 or ICD-10: T67.1), heat cramps (ICD-9-CM: 992.2 or ICD-10: T67.2), heat fatigue (ICD-9-CM: 992.6 or ICD-10: T67.6), and other HRI (ICD-9-CM: 992.3–992.5 or ICD-10: T67.3–T67.5). We obtained variables including age, sex, season, monthly income, comorbidities, concomitant conditions, and medical facilities. We further categorized all the HRI patients into three age groups: 20–44, 45–64, and ≥ 65 years according to the definitions of adulthood, middle-aged persons, and elders by the Taiwanese government [21]. We identified seasons as spring (March, April, and May), summer (June, July, and August), autumn (September, October, and November), and winter (December, January, and February). Monthly income was classified as three subgroups: <20,000, 20,000-3999, and ≥ 40,000 New Taiwan Dollars (NTD). We studied medical comorbidities including hypertension (ICD-9-CM: 401–405 or ICD-10: I10–I16), diabetes (ICD-9-CM: 250 or ICD-10: E08–E13), hyperlipidemia (ICD-9-CM: 272 or ICD-10: E78), cardiovascular disease (ICD-9-CM: 390–398, 410–429, 440–448 or ICD-10: I00–I02, I05–I09, I20, I26–I28, I30–I52, I70–I75, I77–I79), chronic obstructive pulmonary disease (COPD, ICD-9: 490–492, 496 or ICD-10: J40, J410, J411, J418, J42, J430, J431, J432, J438, J439, J440, J441, J449), cerebrovascular disease (ICD-9-CM: 430–438 or ICD-10: I60–I69), renal disease (ICD-9-CM: 580–593 or ICD-10: N00–N20, N25–N29), metal disorder (ICD-9-CM: 290–319 or ICD-10: F01–F99), and malignancy(ICD-9-CM: 140–208 or ICD-10: C00–C69). These nine comorbidities were determined as being diagnosed if the patient had been coded at least three times in ambulatory care claims or once in hospital claims before a diagnosis of HRI. Concomitant conditions included seizure (ICD-9-CM: 345, 780.3 or ICD-10: R56, G40), rhabdomyolysis (ICD-9-CM: 728.88 or ICD-10: M6282), and shock (ICD-9-CM: 785.5 or ICD-10: R57) which coincided with a diagnosis of HRI. We also obtained the data on the types of medical facilities, hospitals or clinics, where HRI patients were treated. Furthermore, we calculated percentage of the HRI patients who visited ED based on the data from ED care and the percentage of the HRI patients who had hospitalizations based on the data from hospital claims. We also assessed 7-day, 1-month, and 3-month mortality rates of HRI, according to a previous study [22]. To ensure data accuracy and minimize potential bias, study subjects with missing data were excluded from the analysis.

### Temperature data and geographic information

We obtained meteorological data on monthly mean temperature from Taiwan’s Central Weather Bureau. Taiwan is divided into 22 subnational divisions, comprising 6 special municipalities (Taipei, New Taipei, Taoyuan, Taichung, Tainan, and Kaohsiung), 13 counties (Hsinchu, Miaoli, Changhua, Nantou, Yunlin, Chiayi, Pingtung, Yilan, Hualien, Taitung, Penghu, Kinmen, and Lienchiang), and 3 cities (Keelung, Hsinchu, and Chiayi). We identified the administrative division that each HRI patient visited and calculated the incidence rate of the division through dividing the number of new incident HRI patients by mid-year population in 2010. Subsequently, we used ArcGIS Version 10.8 (ESRI, Redlands, CA, USA) to map the data, including incidence rate and average monthly mean temperature in each division.

### Data analyses

We described the epidemiological characteristics of HRI patients and evaluated the differences between male and female patients. After performing a normality test, continuous variables such as age were presented as the mean with standard deviation (SD), and Student’s t-test was used to evaluate differences between groups. Categorical variables were expressed as frequencies with percentages, and Pearson’s χ² test was used to assess differences between groups. In addition, we assessed the mortality rates and the distributions of sex and age in different types of HRI. We carried out all analyses using the Statistical Analysis Software Version 9.3 (SAS Inc., Raleigh, NC, USA) at the significant level of 0.05 (two-tailed).

## Results

### Incidence rate of HRI

**Fig. 1:**
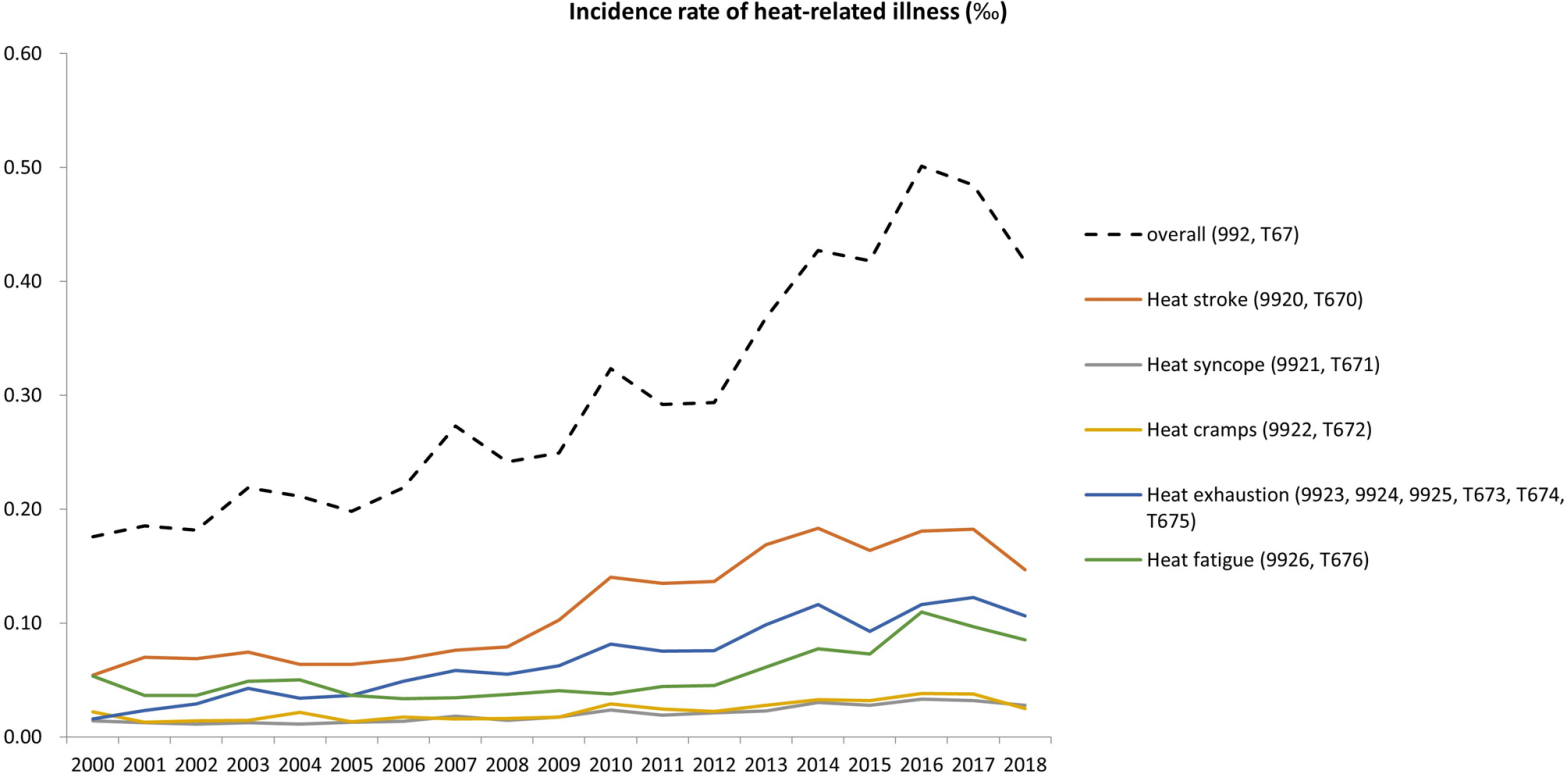
Incidence rates of overall and different types of heat-related illness in Taiwan between 2000 and 2018

Between 2000 and 2018, there were a total of 101,614 patients experiencing HRI events. The incidence rates of HRI increased gradually, ranging from 1.76 per 10,000 population in 2000, to 4.17 per 10,000 population in 2018 (Fig. 1). In different types of HRI, heat stroke had the highest incidence rate, followed by heat exhaustion (Fig. 1).

### Descriptive analysis of the HRI patients

Of the 101,614 HRI patients, 56.2% were males, and the mean age was 48.2 ± 18.0 years, with most of the patients in the age groups of 20–44 years (44.8%). Most of the HRI occurred in summer (67.9%) and in those with monthly income less than 20,000 NTD (65.9%). As to medical comorbidities, 22.2% of the HRI patients had hypertension, 12.3% had mental disorder, and 10.0% had hyperlipidemia. Less than 1% of the HRI patients had concomitant conditions such as seizure, rhabdomyolysis, or shock. Nearly 60% of the HRI patients sought medical care in clinics (Table 1). The proportion of the HRI patients who had visited an ED care was 22.2%, and only 4.9% of all HRI patients were hospitalized.

**Table 1:** Demographic characteristics, comorbidities, concomitant conditions, medical facilities and dispositions in patients with heat-related illness in Taiwan between 2000 and 2018

	Total			Male			Female		
	*N*	%		*N*		%	*N*	%	*p* value^a^
Cases	101,614	100		57,131		56.2	44,483	43.8	
Age (year)									< 0.001
mean ± SD	48.2 ± 18.0			46.4 ± 18.1			50.5 ± 17.6		
Age group (year)									< 0.001
20–44	45,583		44.8	28,292		49.5	17,291	38.9	
45–64	34,317		33.8	17,943		31.4	16,374	36.8	
≥ 65	21,714		21.4	10,896		19.1	10,818	24.3	
Season^b^									< 0.001
Spring	15,572		15.3	8773		15.4	6799	15.3	
Summer	68,977		67.9	38,772		67.9	30,205	67.9	
Fall	13,581		13.4	7969		14.0	5612	12.6	
Winter	3484		3.4	1617		2.8	1867	4.2	
Monthly income^c^									< 0.001
< 20,000 NTD	63,868		65.9	35,563		65.9	28,305	66.0	
20,000–39,999 NTD	22,062		22.8	11,642		21.6	10,420	24.3	
≥ 40,000 NTD	10,957		11.3	6784		12.6	4173	9.7	
Comorbidity									
Hypertension	22,577		22.2	11,699		20.5	10,878	24.5	< 0.001
Diabetes	10,010		9.9	5179		9.1	4831	10.9	< 0.001
Hyperlipidemia	10,124		10.0	5094		8.9	5030	11.3	< 0.001
Cardiovascular disease	9293		9.1	5026		8.8	4267	9.6	< 0.001
COPD	3360		3.3	2267		4.0	1093	2.5	< 0.001
Cerebrovascular disease	3679		3.6	2243		3.9	1436	3.2	< 0.001
Renal disease	5875		5.8	4204		7.4	1671	3.8	< 0.001
Mental disorder	12,452		12.3	6060		10.6	6392	14.4	< 0.001
Malignancy	2254		2.2	1175		2.1	1079	2.4	< 0.001
Concomitant condition									
Seizure	635		0.6	545	1.0		90	0.2	< 0.001
Rhabdomyolysis	421		0.4	367	0.6		54	0.1	< 0.001
Shock	324		0.3	256	0.5		68	0.2	< 0.001
Medical facility^d^									< 0.001
Hospital	40,705		40.1	29,460	51.6		11,245	25.3	
Clinic	60,907		59.9	27,670	48.4		33,237	74.7	

*SD* standard deviation, *NTD* New Taiwan Dollars, *COPD* chronic obstructive pulmonary disease

^a^Comparison between males and females

^b^Spring: March, April, and May; summer: June, July, and August; autumn: September, October, and November; winter: December, January, and February

^c^Not all the patients had information on monthly income

^d^Not all the patients had information on medical facility

### Comparisons between male and female patients

In comparison with female patients, male patients were younger (46.4 vs. 50.5 years, *p* < 0.001) and had higher proportions of having seizure (1.0% vs. 0.2%, *p* < 0.001), rhabdomyolysis (0.6% vs. 0.1%, *p* < 0.001), and shock (0.5% vs. 0.2%, *p* < 0.001). Male patients had a lower prevalence of all medical comorbidities evaluated, except for COPD, cerebrovascular diseases, and renal diseases. Additionally, male patients were more likely to receive treatment in hospitals (51.6% vs.25.3%, *p* < 0.001) (Table 1).

### Sex, age, and mortality rates in different types of HRI

**Fig. 2:**
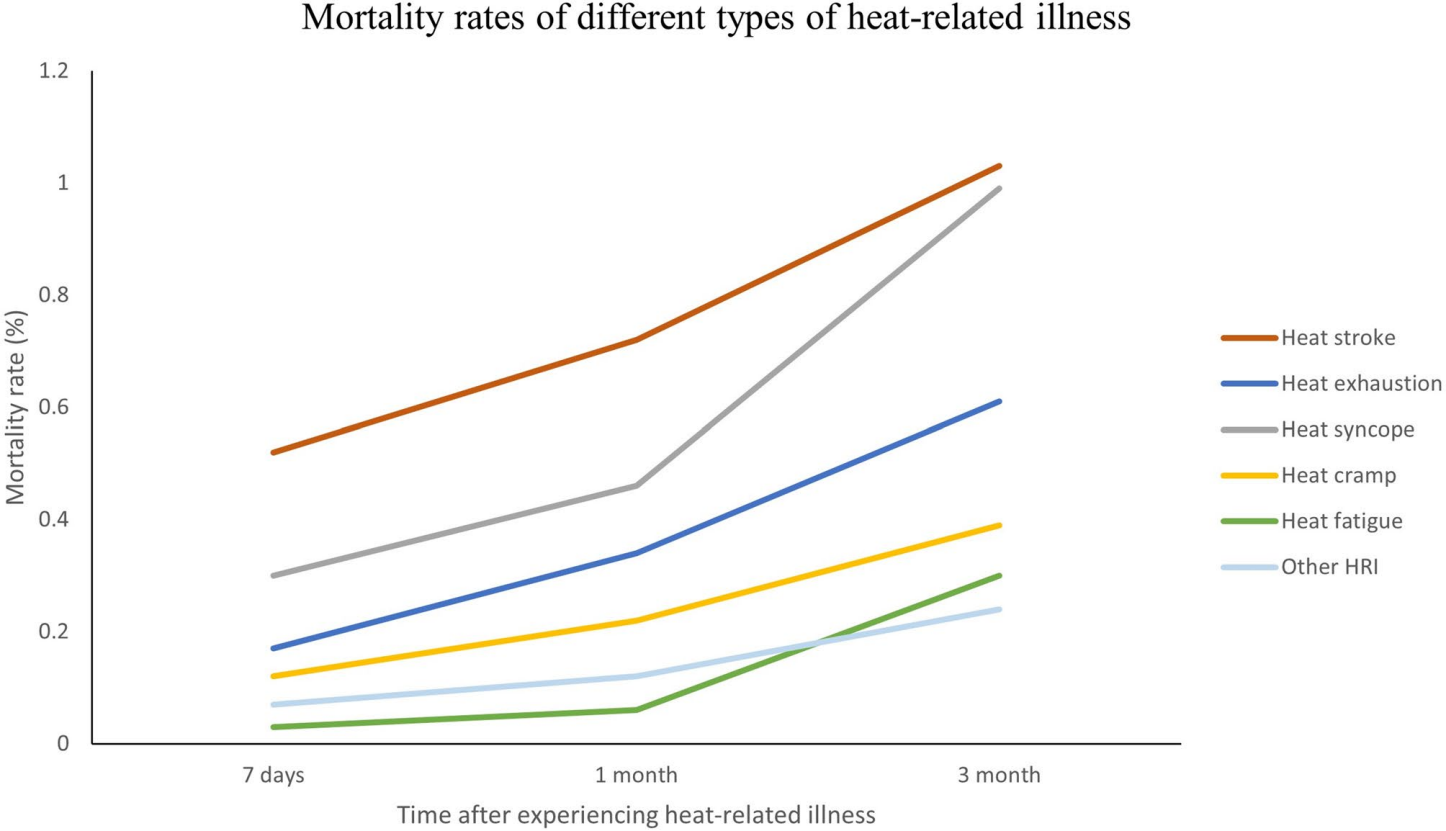
The 7-day, 1-month, and 3-month mortality rates of different types of heat-related illness

In all types of HRI, most of the patients were male except for “other HRI.” Most of the patients were in the age groups of 20–44 years across different diagnoses (Table 2). Among the diagnoses, heat stroke had the highest mortality rate (Fig. 2). The 7-day, 1-month, and 3-month mortality rates in heat stroke patients were 0.5%, 0.7% and 1.0%, respectively (Table 2).

**Table 2:** Sex, age and mortality in different types of heat-related illness

	Heat stroke		Heat exhaustion		Heat syncope		Heat cramp		Heat fatigue		Other HRI	
	*N*	%	*N*	%	*N*	%	*N*	%	*N*	%	*N*	%
*Total*	38,752	100.0	23,309	100.0	6753	100.0	7765	100.0	18,546	100.0	10,809	100.0
*Sex*												
Male	21,513	55.5	15,049	64.6	4148	61.4	5574	71.8	9506	51.3	4384	40.6
Female	17,239	44.5	8260	35.4	2605	38.6	2191	28.2	9040	48.7	6425	59.4
*Age groups*												
20–44	15,400	39.7	11,306	48.5	2740	40.6	4288	55.2	7999	43.1	5713	52.9
45–64	13,860	35.8	7534	32.3	2223	32.9	2596	33.4	6205	33.5	3378	31.3
≥65	9492	24.5	4469	19.2	1790	26.5	881	11.4	4342	23.4	1718	15.9
*Mortality*												
7 days	200	0.52	39	0.17	20	0.30	9	0.12	5	0.03	8	0.07
1 month	278	0.72	80	0.34	31	0.46	17	0.22	12	0.06	13	0.12
3 months	400	1.03	143	0.61	67	0.99	30	0.39	55	0.30	26	0.24

### Geographic distributions of HRI

**Fig. 3:**
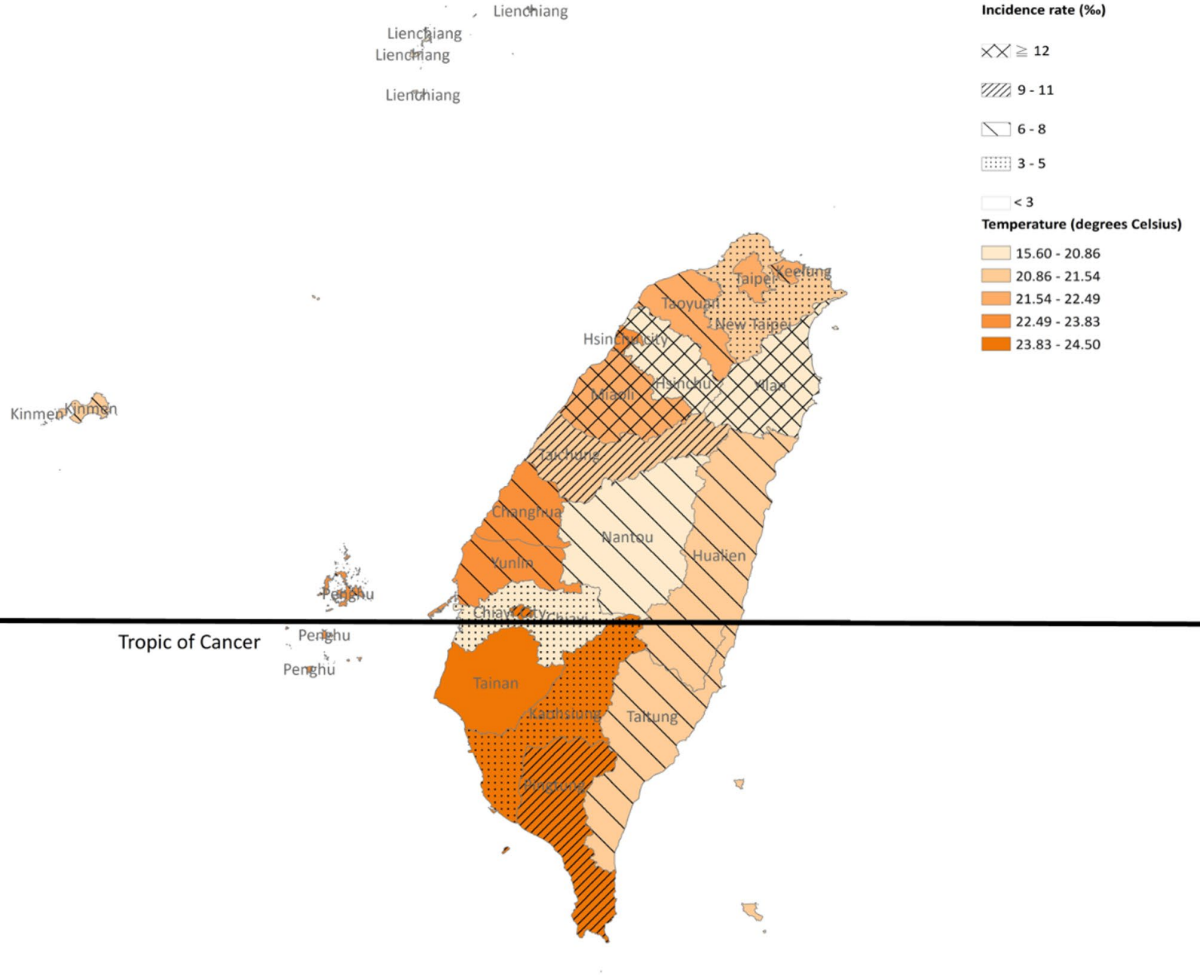
Geographic distribution of incident heat-related illness between 2000 and 2018. The color was for temperature gradient, and lines or dots were for levels of incidences rates by division

Geographic distribution showed that the incident HRI patients were observed more in Hsinchu, Miaoli, and Yilan that located in the subtropical zone (Fig. 3).

## Discussion

This nationwide study described the epidemiological characteristics of HRI in Taiwan between 2000 and 2018. The incidence of HRI increased gradually over a 19-year period. The majority of HRI patients were male and young. Male patients were younger and more likely to receive treatment in hospitals than female patients. Most of the HRI patients sought medical care in clinics. Among the different types of HRI, heat stroke had the highest incidence rate and mortality rate.

Previous nationwide studies on the incidence of HRI were limited to specific populations of interest, such as patients with ED visits [16, 17], high school athletes [14], and military personnel [15]. In contrast, this study described the epidemiological characteristics of HRI in a general population and revealed that the incidence rates of HRI increased gradually, ranging from 1.76 per 10,000 population in 2000, to 4.17 per 10,000 population in 2018. The results confirmed the concern of an increased incidence of HRI under the scenario of global warming.

This study showed that there were more male HRI patients than female HRI patients, which is consistent with previous studies [16, 17, 23, 24]. A systematic review investigating the impact of sex on occurrence of HRI across the lifespan found that males had a significantly higher risk of HRI compared to females. The reasons for this difference may include physiological, psychological, and behavioral factors [25]. Studies have shown that females were more likely to report symptoms in a heat event than males [26, 27], while males were more likely to experience more severe HRI than females [25]. In our study, we found that male patients had a higher percentage of concomitant conditions and were more likely to receive treatment in hospitals compared to female patients, indicating that male patients might be in more severe conditions than female patients when they seek for medical care for HRI. However, in different types of HRI, the sex difference varied, from 71.8% males in the heat cramp group to 51.3% in the heat fatigue group. The results provide an implication that preventive measures taken for HRI should be more focus on male.

Regarding other demographic characteristics in this study, the mean age for HRI patients was 48.2 years with majority of the patients in the 20 to 64 age group (44.8% in 20–44 age group, and 33.8% in 45–64 age group), which was similar to a study describing Emergency Medical Services activation for HRI in the United States [28]. In different types of HRI, this study showed that the highest proportion of HRI cases occurred in the 20–44 age group across all HRI subtypes. This aligned with previous research suggesting that occupational exposure, outdoor physical activity, and lifestyle factors are major contributors to HRI risk in this age group [18, 28, 29]. We also found that male HRI patients tend to be younger than female patients, which might be attributed to several factors. Younger males are more likely to engage in high-risk occupations such as construction, agriculture, and manual labor, which involve prolonged outdoor exposure to heat and physically demanding activities [18, 30]. Behavioral factors also play a role, as younger males had lower risk awareness and underestimated the dangers of heat exposure and delay preventive measures, such as hydration or seeking shade [25]. These findings underscore the need for targeted interventions, including workplace safety policies, education on heat risks, and awareness campaigns, to reduce HRI risk in younger males. In terms of socioeconomic status, people with lower socioeconomic status or those living in lower income household were found to be at increased risk for HRI [4, 29]. Our study found that most of the HRI patients were in the lowest income group (65.9%), which was consistent with previous studies.

In this study, a very small proportion of HRI patients had concomitant conditions such as seizure, rhabdomyolysis, or shock, and only 4.9% of HRI patients were hospitalized. Previous studies using national ED database in the United States demonstrated that 6.5–12% of HRI patients were admitted to hospitals [12, 16, 24]. The difference might be attributable to differences in the health care system, geographic region, etc. However, it still showed that most of HRI patients were treated and released without complicated situations, either in the ED or in the general population. We also found that nearly 60% of HRI patients sought medical care in clinics. The healthcare system in Taiwan is characterized by good accessibility and comprehensive population coverage [31]. Patients have easy access to clinics with low co-payment and short waiting times [32]. Since the majority of HRI can be benign and reversed [10], clinics should be a suitable medical facility for treatment in most cases.

Our study showed that heat stroke was the most common type of HRI, which was different from the previous studies. Most previous studies revealed that heat exhaustion was the most common type of HRI in the ED [12, 16, 24]. To date, the diagnosis of HRI still relies on clinical presentations, without a single diagnostic test to confirm it [10]. Clinically, heat stroke is characterized by a core temperature exceeding 40 °C with central nerve system dysfunction [4]. Early neurological manifestations of heat stroke may include dizziness, weakness, nausea, vomiting, and confusion [4, 33]. It is generally advised that any changes in mental status in patients with heat exhaustion must be considered as heat stroke regardless of the core temperature [9]. As heat exhaustion may progress to heat stroke, and early neurological presentations are nonspecific, it may be difficult to differentiate between these two diagnoses especially in the early stage of clinical manifestations [33]. Therefore, a portion of heat exhaustion cases might be categorized as heat stroke. In addition, during hot weather, the Taiwanese government frequently advocated for instructions and educations on heat stroke [34], which may raise the awareness for people experiencing HRI to seek medical care early and for clinical physicians with a high index of suspicion in diagnosing heat stroke.

Regarding the geographic distributions of HRI, we found that the incident rate was higher in Hsinchu, Miaoli, and Yilan that located in the subtropical region rather than in the tropical region. It indicated that the occurrence of HRI was not merely related to ambient temperature. Other factors such as geographic and climate factors, occupational exposure, availability and usage of air conditioning, and acclimatization, may also contribute to the occurrence of HRI [16, 24].

An obvious strength of this study was its investigation of the epidemiological characteristics of HRI in a general population, which distinguished it from previous studies. We believe that the results of this study can provide additional knowledge on HRI. However, this study still had some limitations. First, the diagnosis of HRI was based solely on diagnostic codes and misclassification bias may exist. It is possible that a clinical physician may choose the diagnostic code of heat stroke, instead of heat exhaustion, for a patient with severe symptoms after heat exposure but not really meeting the strict criteria of heat stroke. This might lead to an overestimate of heat stroke. Second, the NHIRD did not provide information on the etiology of HRI, body mass index, alcohol drinking habits, and other sociodemographic factors such as education level, Therefore, we could not investigate these factors in this study. Third, we only obtained data on ambient temperature and the incident HRI patients for mapping the geographic distribution of HRI and did not have information on other factors such as usage of air conditioning and occupational exposure. Likewise, we could not investigate these factors in this study. Fourth, the results in this study may not be generalizable to other nations due to the differences in contributing factors such as climate, health care system, and culture.

## Conclusions

In Taiwan, HRI patients are more likely to be male and between 20 and 44 years old. Male patients were younger and more likely to receive treatment in hospitals. Heat stroke was the most common type of HRI and had the highest mortality rate, which highlights the need for the establishment of prevention and treatment strategies.

## Data Availability

The data supporting the findings of this study were obtained from the Taiwan National Health Insurance Research Database, managed by the Health and Welfare Data Science Center (HWDC), Ministry of Health and Welfare, Taiwan. Due to legal, ethical, and privacy restrictions, these data are publicly available to Taiwanese researchers. Requests for data access can be submitted as formal proposals to the HWDC (https://dep.mohw.gov.tw/DOS/cp-5283-63826-113.html), and payment is required.

## Abbreviations

HRI: Heat-related illness
ED: Emergency department
CDC: Center for Disease Control and Prevention
NHIRD: National Health Insurance Research Database
NHI: National Health Insurance
ICD-9-CM: International Classification of Diseases, Ninth Revision, Clinical Modification
ICD-10: International Classification of Diseases, Tenth Revision
NTD: New Taiwan Dollars
COPD: Chronic obstructive pulmonary disease

## References

[1] Vicedo-Cabrera AM, Scovronick N, Sera F, Royé D, Schneider R, Tobias A, et al. The burden of heat-related mortality attributable to recent human-induced climate change. Nat Clim Chang. 2021;11(6):492–500. 10.1038/s41558-021-01058-x. 34221128 10.1038/s41558-021-01058-x PMC7611104

[2] Watts N, Amann M, Arnell N, Ayeb-Karlsson S, Beagley J, Belesova K, et al. The 2020 report of the lancet countdown on health and climate change: responding to converging crises. Lancet. 2021;397(10269):129–70. 10.1016/s0140-6736(20)32290-x. 33278353 10.1016/S0140-6736(20)32290-X PMC7616803

[3] Vaidyanathan A, Gates A, Brown C, Prezzato E, Bernstein A. Heat-related emergency department visits - United States, May-September 2023. MMWR Morb Mortal Wkly Rep. 2024;73(15):324–9. 10.15585/mmwr.mm7315a1. 38635484 10.15585/mmwr.mm7315a1 PMC11037437

[4] Sorensen C, Hess J. Treatment and prevention of heat-related illness. N Engl J Med. 2022;387(15):1404–13. 10.1056/NEJMcp2210623. 36170473 10.1056/NEJMcp2210623

[5] Leiva DF, Church B, Heat illness. 2023 Apr 10 In: StatPearls. Treasure Island (FL): StatPearls Publishing; 2024. PMID: 31971756. 31971756

[6] Eifling KP, Gaudio FG, Dumke C, Lipman GS, Otten EM, Martin AD, et al. Wilderness medical society clinical practice guidelines for the prevention and treatment of heat illness: 2024 update. Wilderness Environ Med. 2024;35(1suppl):s112–27. 10.1177/10806032241227924. 10.1177/10806032241227924 38425235

[7] Epstein Y, Yanovich R, Heatstroke. N Engl J Med. 2019;380(25):2449–59. 10.1056/NEJMra1810762. 31216400 10.1056/NEJMra1810762

[8] Cheshire WP. Jr. Thermoregulatory disorders and illness related to heat and cold stress. Auton Neurosci. 2016;196:91–104. 10.1016/j.autneu.2016.01.001. 26794588 10.1016/j.autneu.2016.01.001

[9] Gauer R, Meyers BK. Heat-related illnesses. Am Fam Physician. 2019;99(8):482–9. 30990296

[10] Santelli J, Sullivan JM, Czarnik A, Bedolla J. Heat illness in the emergency department: keeping your cool. Emerg Med Pract. 2014;16(8):1–21. quiz – 2. 25422847

[11] Atha WF. Heat-related illness. Emerg Med Clin North Am. 2013;31(4):1097–108. 10.1016/j.emc.2013.07.012 24176481 10.1016/j.emc.2013.07.012

[12] Sanchez CA, Thomas KE, Malilay J, Annest JL. Nonfatal natural and environmental injuries treated in emergency departments, United States, 2001–2004. Fam Community Health. 2010;33(1):3–10. 10.1097/FCH.0b013e3181c4e2fa 20010000 10.1097/FCH.0b013e3181c4e2fa

[13] QuickStats. Percentage Distribution of Heat-related Deaths,* by Age Group - National Vital Statistics System, United States, 2018–2020. MMWR Morb Mortal Wkly Rep. 2022;71(24):808. 10.15585/mmwr.mm7124a6 10.15585/mmwr.mm7124a6 35709072

[14] Kerr ZY, Casa DJ, Marshall SW, Comstock RD. Epidemiology of exertional heat illness among U.S. high school athletes. Am J Prev Med. 2013;44(1):8–14. 10.1016/j.amepre.2012.09.058 23253644 10.1016/j.amepre.2012.09.058

[15] Williams VF, Oh GT. Update: Heat illness, active component, US Armed Forces, 2021. MSMR. 2022;29(4):8–14. 35608520

[16] Hess JJ, Saha S, Luber G. Summertime acute heat illness in U.S. emergency departments from 2006 through 2010: analysis of a nationally representative sample. Environ Health Perspect. 2014;122(11):1209–15. 10.1289/ehp.1306796 24937159 10.1289/ehp.1306796 PMC4216158

[17] Dring P, Armstrong M, Alexander R, Xiang H. Emergency department visits for heat-related emergency conditions in the United States from 2008–2020. Int J Environ Res Public Health. 2022;19(22). 10.3390/ijerph192214781 10.3390/ijerph192214781 PMC9690248 36429500

[18] Kakamu T, Endo S, Hidaka T, Masuishi Y, Kasuga H, Fukushima T. Heat-related illness risk and associated personal and environmental factors of construction workers during work in summer. Sci Rep. 2021;11(1):1119. 10.1038/s41598-020-79876-w. 33441683 10.1038/s41598-020-79876-w PMC7806839

[19] National Health Insurance Administration. Ministry of Health and Welfare, Taiwan, ROC. National Health Insurance Annual Report 2014–2015. Ministry of Health and Welfare Taipei, Taiwan. 2014.

[20] Hsieh CY, Su CC, Shao SC, Sung SF, Lin SJ, Kao Yang YH, et al. Taiwan’s National health insurance research database: past and future. Clin Epidemiol. 2019;11:349–58. 10.2147/clep.S196293. 31118821 10.2147/CLEP.S196293 PMC6509937

[21] Ministry of Labor, Taiwan ROC. Middle-aged and Elderly Employment Promotion Act 2019.

[22] Hausfater P, Megarbane B, Dautheville S, Patzak A, Andronikof M, Santin A, et al. Prognostic factors in non-exertional heatstroke. Intensive Care Med. 2010;36(2):272–80. 10.1007/s00134-009-1694-y. 19841896 10.1007/s00134-009-1694-y

[23] Kalaiselvan MS, Renuka MK, Arunkumar AS. A retrospective study of clinical profile and outcomes of critically ill patients with heat-related illness. Indian J Anaesth. 2015;59(11):715–20. 10.4103/0019-5049.170030. 26755836 10.4103/0019-5049.170030 PMC4697243

[24] Pillai SK, Noe RS, Murphy MW, Vaidyanathan A, Young R, Kieszak S, et al. Heat illness: predictors of hospital admissions among emergency department visits-Georgia, 2002–2008. J Community Health. 2014;39(1):90–8. 10.1007/s10900-013-9743-4. 23934476 10.1007/s10900-013-9743-4

[25] Gifford RM, Todisco T, Stacey M, Fujisawa T, Allerhand M, Woods DR, et al. Risk of heat illness in men and women: A systematic review and meta-analysis. Environ Res. 2019;171:24–35. 10.1016/j.envres.2018.10.020. 30641370 10.1016/j.envres.2018.10.020

[26] Bélanger D, Gosselin P, Valois P, Abdous B. Perceived adverse health effects of heat and their determinants in deprived neighbourhoods: a cross-sectional survey of nine cities in Canada. Int J Environ Res Public Health. 2014;11(11):11028–53. 10.3390/ijerph111111028. 25347192 10.3390/ijerph111111028 PMC4245598

[27] Nitschke M, Hansen A, Bi P, Pisaniello D, Newbury J, Kitson A, et al. Risk factors, health effects and behaviour in older people during extreme heat: a survey in South Australia. Int J Environ Res Public Health. 2013;10(12):6721–33. 10.3390/ijerph10126721. 24300073 10.3390/ijerph10126721 PMC3881137

[28] Yeargin S, Hirschhorn R, Grundstein A. Heat-related illnesses transported by United States emergency medical services. Medicina (Kaunas). 2020;56(10). 10.3390/medicina56100543 10.3390/medicina56100543 PMC7602997 33080867

[29] Gronlund CJ. Racial and socioeconomic disparities in heat-related health effects and their mechanisms: a review. Curr Epidemiol Rep. 2014;1(3):165–73. 10.1007/s40471-014-0014-4. 25512891 10.1007/s40471-014-0014-4 PMC4264980

[30] Spector JT, Masuda YJ, Wolff NH, Calkins M, Seixas N. Heat exposure and occupational injuries: review of the literature and implications. Curr Environ Health Rep. 2019;6(4):286–96. 10.1007/s40572-019-00250-8. 31520291 10.1007/s40572-019-00250-8 PMC6923532

[31] Wu TY, Majeed A, Kuo KN. An overview of the healthcare system in Taiwan. Lond J Prim Care (Abingdon). 2010;3(2):115–9. 10.1080/17571472.2010.11493315. 10.1080/17571472.2010.11493315 PMC3960712 25949636

[32] Shao CC, Chang CP, Chou LF, Chen TJ, Hwang SJ. The ecology of medical care in Taiwan. J Chin Med Assoc. 2011;74(9):408–12. 10.1016/j.jcma.2011.08.005. 21962249 10.1016/j.jcma.2011.08.005

[33] Leon LR, Bouchama A. Heat stroke. Compr Physiol. 2015;5(2):611–47. 10.1002/cphy.c140017. 25880507 10.1002/cphy.c140017

[34] Huang TT. Men in Taiwan urged to block sun with umbrellas as heatstroke cases surge. Accessed 27 Feb 2023. https://www.taiwannews.com.tw/en/news/3965718

